# Regular Exercise Decreases the Risk of Osteoporosis in Postmenopausal Women

**DOI:** 10.3389/fpubh.2022.897363

**Published:** 2022-06-15

**Authors:** Chu-Fen Chang, Jia-In Lee, Shu-Pin Huang, Jiun-Hung Geng, Szu-Chia Chen

**Affiliations:** ^1^Department of Physical Therapy, Tzu Chi University, Hualien, Taiwan; ^2^Department of Psychiatry, Kaohsiung Medical University Hospital, Kaohsiung Medical University, Kaohsiung, Taiwan; ^3^Department of Urology, Kaohsiung Medical University Hospital, Kaohsiung Medical University, Kaohsiung, Taiwan; ^4^Department of Urology, Faculty of Medicine, College of Medicine, Kaohsiung Medical University, Kaohsiung, Taiwan; ^5^Graduate Institute of Clinical Medicine, College of Medicine, Kaohsiung Medical University, Kaohsiung, Taiwan; ^6^Ph.D. Program in Environmental and Occupational Medicine, College of Medicine, Kaohsiung Medical University, Kaohsiung, Taiwan; ^7^Research Center for Environmental Medicine, Kaohsiung Medical University, Kaohsiung, Taiwan; ^8^Department of Urology, Kaohsiung Municipal Siaogang Hospital, Kaohsiung, Taiwan; ^9^Department of Internal Medicine, Kaohsiung Municipal Siaogang Hospital, Kaohsiung Medical University, Kaohsiung, Taiwan; ^10^Division of Nephrology, Department of Internal Medicine, Kaohsiung Medical University Hospital, Kaohsiung Medical University, Kaohsiung, Taiwan; ^11^Faculty of Medicine, College of Medicine, Kaohsiung Medical University, Kaohsiung, Taiwan

**Keywords:** osteoporosis, exercise, postmenopausal women, epidemiologic study, risk factors

## Abstract

Regular exercise can regulate bone maintenance and improve bone health. However, large-scale epidemiological studies on the association between regular exercise and incident osteoporosis in menopausal women are still lacking. We aimed to examine the relationship between exercise and the risk of osteoporosis in menopausal women. In cross-sectional analysis, we enrolled 30,046 postmenopausal women with available information from the database of the Taiwan Biobank (TWB). We divided them into two groups according to their status of regular exercise, i.e., no exercise and regular exercise groups. A *t*-score of −2.5 or more standard deviations (SDs) below that of a young adult was defined as osteoporosis. Logistic regression after adjusting for confounding factors was used to analyze the association between regular exercise and the prevalence of osteoporosis. Furthermore, the risk of incident osteoporosis development was analyzed in a longitudinal cohort of 6,785 postmenopausal women without osteoporosis at baseline using a Kaplan-Meier analysis and a log-rank test. The mean age of subjects in the cross-sectional cohort was 59 years old. Fifty-six percent of them were exercising regularly. Osteoporosis was observed in 1,886 (14.2%) and 2,254 (13.4%) participants in the no exercise and regular exercise groups. Lower risk of osteoporosis was noted in postmenopausal women with regular exercise when compared with those without regular exercise [odds ratio (OR), 0.76; 95% confidence interval (95% CI), 0.71–0.81]. In the longitudinal cohort, incident osteoporosis was found in 430 (10.5%) women with regular exercise and 299 (11.2%) women without exercise during a mean follow-up of 45 months. Cox regression analysis revealed that the risk for incident osteoporosis was lower in postmenopausal women with regular exercise than those without exercise [hazard ratio (HR), 0.83; 95% CI, 0.71–0.97]. Our study suggests that regular exercise is associated with a reduced risk of osteoporosis in postmenopausal women and strengthens the importance of exercise for the prevention of osteoporosis.

## Introduction

Menopause is a biological transition marking the complete cessation of menstrual cycles for 12 consecutive months with a decline in ovarian hormone production, and the median age of natural menopause was 51.3 years (1). In 2020, the average life expectancy of Taiwanese women has increased to 84.75 years (2), indicating menopause occupies more than one-third of women's lifespan and its impact on women's health unarguably is a major public health concern. The possible consequences of the effects of menopause have been indicated to be related to not only increased risks of significant psychological health conditions, such as depression, dementia, and schizophrenia, but also increased morbidity of significant medical conditions, such as osteoporosis (and subsequent fractures), cardiovascular disease, type 2 diabetes, and some types of cancers, such as breast cancer (1, 3–6).

Osteoporosis is the most prevalent systemic skeletal disease worldwide, affecting ~30% of all postmenopausal women in the United States and Europe (7, 8). This progressive osteometabolic disease is characterized by the deterioration of bone microarchitecture and substantial decrease in bone mass, predisposing to compromised bone strength, increased bone fragility, and a consequent increased risk of fracture occurring with low-energy trauma (9, 10). Osteoporosis is also commonly known as a silent skeletal disease because it has no obvious manifestations until low-impact fractures due to bone fragility occur (11). Among postmenopausal women with osteoporosis, at least 40% of them suffer from one or more osteoporotic fractures in their remaining life span (7, 8). Osteoporotic fracture is extremely harmful to postmenopausal women because it frequently results in debilitating pain and physical disability, leading to further loss of independence, deterioration of health-related quality of life, depression, high risk of mortality, and even premature deaths (12–14). The annual cost of osteoporosis to health systems is also enormous, estimated to be $25.3 billion by 2025 (15). Additionally, it is regrettable that the available treatment options are not very effective once osteoporosis presents (3). Therefore, the establishment of effective strategies for the prevention of debilitating osteoporosis and succeeding fractures should be the priority.

Preventive and ameliorative strategies for osteoporosis include regular physical activity (particularly weight-bearing exercise), adequate nutrition (particularly calcium together with vitamin D intake), avoiding poor living habits that have adverse effects on bone health (such as tobacco use and alcohol intake), and hormone replacement therapy that has been proposed to maintain and achieve peak bone mass (3, 16). Among the recommended strategies, regular physical exercise has been indicated to have beneficial effects on bone health in older women and has been considered the most powerful non-pharmaceutical strategy to prevent osteoporotic fracture in postmenopausal women (17, 18). There were also randomized trials demonstrating that exercise could preserve bone mineral density (BMD) in postmenopausal women (19, 20), but the majority of these studies were small. Large-scale epidemiological studies on the association between regular physical exercise and incident osteoporosis in menopausal women are still lacking, so our study is aimed to investigate the relationship between physical exercise and the risk of osteoporosis in menopausal women.

## Materials and Methods

### Subjects

The study subjects in the present study were postmenopausal women collected from Taiwan Biobank (TWB). Women were considered to be postmenopausal when their menstrual period had been gone for longer than 1 year. TWB is a population-based biobank in Taiwan where more than 100,000 participants aged between 30 and 70 years were recruited since 2008. It includes information regarding medical history, environmental exposure, lifestyles, physical examinations, BMD, and blood tests. The majority of participants were cancer free (over 99%) and belonged to Han Chinese. Other detailed information about TWB can be found in previous studies (21, 22). On this basis, we aimed to utilize data from TWB to evaluate the association between regular exercise and the risk of osteoporosis in postmenopausal women.

**Figure 1:**
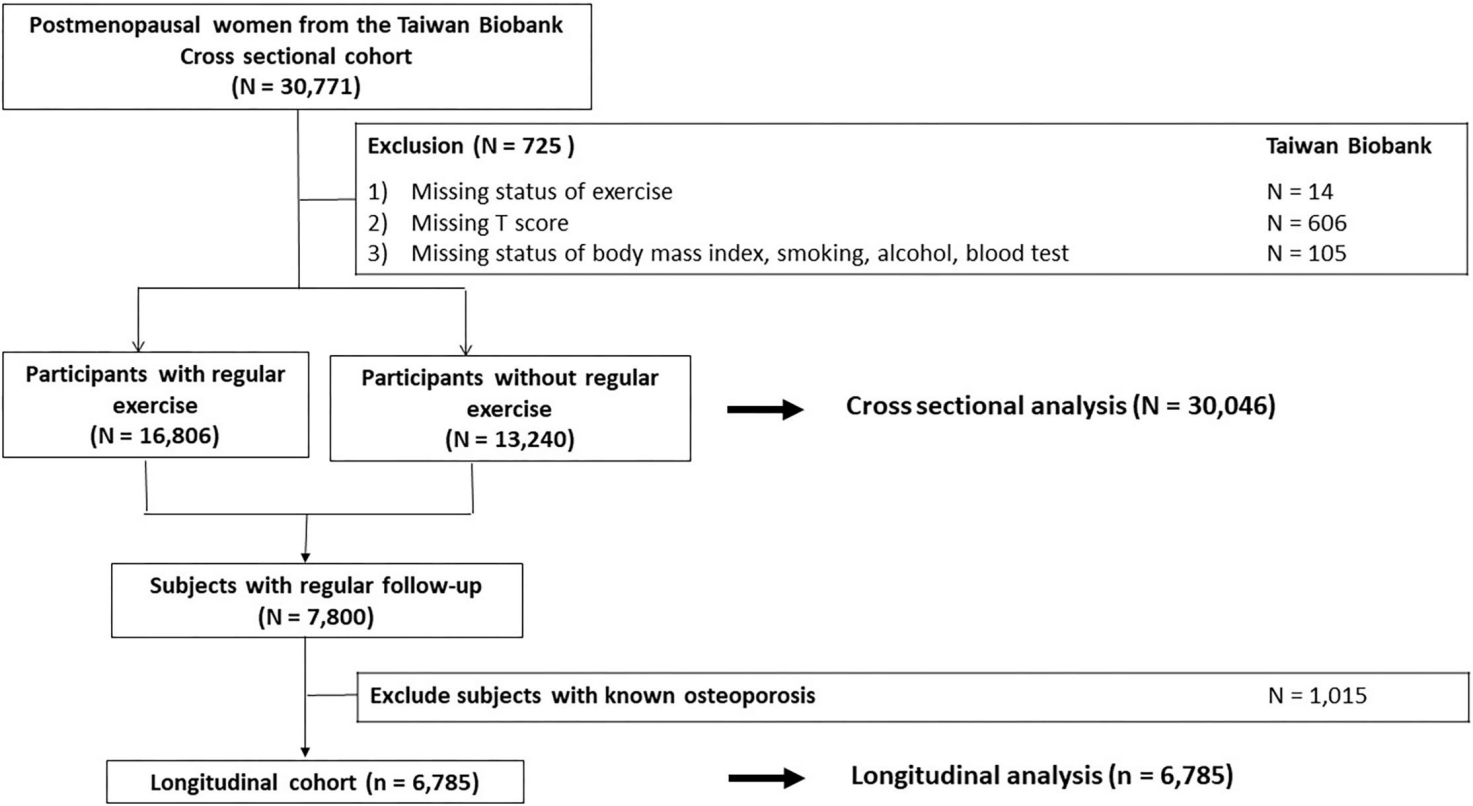
Study participants were classified by the status of regular exercise.

Firstly, a total of 30,771 postmenopausal women are enrolled, as shown in Figure 1, to explore the association between regular exercise and the prevalence of osteoporosis. Individuals with missing information about status of regular exercise (*N* = 14), t-scores (*N* = 606), body mass index [BMI] (*N* = 10), smoking status (*N* = 10), alcohol status (*N* = 18), status of marriage (*N* = 26), status of education (*N* = 10), and blood tests (*N* = 31) were excluded. There were 30,046 postmenopausal women in the final analysis. For these women, data on exercise and *t*-scores were collected at one point in time and were analyzed in a cross-sectional fashion.

Additionally, a total of 7,800 postmenopausal women who received regular follow-up on TWB were enrolled in the longitudinal cohort to examine the association between regular exercise and the development of incident osteoporosis. We excluded those with known osteoporosis at baseline (*N* = 1,015), and a total of 6,785 women were analyzed (Figure 1). Participants in the longitudinal cohort underwent serial questionnaires, BMD tests, and blood tests every 2–4 years from 2008 to 2019. All participants were followed from the date of enrollment until the development of osteoporosis or the end of follow-up (31 December 2019), whichever event came first.

### Ethics Statement

The Institutional Review Board of Kaohsiung Medical University Hospital (KMUHIRB-E(I)-20210058) has approved this study. The written informed consents were provided by all participants, and the Declaration of Helsinki was followed by all the investigators.

### Baseline Characteristics, Medical, and Laboratory Measurements

Baseline variables from the study cohort included age, age of menopause, BMI, systolic blood pressure, diastolic blood pressure, hormone replacement therapy, history of hypertension, diabetes mellitus (DM), dyslipidemia, gout, status of regular exercise, smoking, alcohol (referring to at least 150 ml per week for 6 months), marriage, education, BMD *t*-scores of the heel calcaneus, and laboratory data [white blood counts, red blood counts, platelet counts, hemoglobin, albumin, fasting glucose, hemoglobin A1c, total cholesterol, triglycerides, high-density lipoprotein (HDL) cholesterol, low-density lipoprotein (LDL) cholesterol, creatinine, and uric acid].

### Exercise Status Assessment

Firstly, subjects were asked the following question: “Do you have a habit of regular exercise (referring to at least three times a week, ≧ 10 min for each exercise, and referring to sports, such as walking, running, boxing, or dancing; behavior or labor [farming, housework, etc.] does not include)?” Participants who have a habit of regular exercise were assigned to the regular exercise group; others were allocated to the no exercise group. Participants in the regular exercise group were further asked “How many hours do you exercise each time?” According to the time of each exercise, we divided participants into “no exercise,” “≦1 h,” and “>1 h.”

### Definition of Osteoporosis

In the present study, we used ultrasound (Achilles InSight, GE, USA) to evaluate the estimating BMD of the heel calcaneus. The *t*-score was calculated according to the following formula: [(individual's BMD—mean BMD in young adults)/SD of a normal young-adult population] (23). A *t*-score of −2.5 or more SDs below the young adult were defined as osteoporosis (23). The study outcome was the presence of osteoporosis based on this definition.

### Statistical Analyses

In the present study, participants were stratified into a no exercise group and a regular exercise group. Clinical characteristics were shown as percentages for categorical variables and mean ± SD for continuous variables. For comparison between no exercise and regular exercise groups, chi-square tests for categorical variables and independent *t*-tests for continuous variables were performed. In the cross-sectional cohort, univariate and multivariate logistic regression analyses adjusted for potential confounders (age, BMI, smoking status, education status, systolic blood pressure, diastolic blood pressure, white blood counts, red blood counts, platelet counts, hemoglobin, triglyceride, LDL cholesterol, HDL cholesterol, serum albumin, and serum uric acid) (24–28) were used to assess the association between exercise and the prevalence of osteoporosis. In addition, to examine the relationship between exercise and incident osteoporosis, a subgroup of 6,785 postmenopausal women without osteoporosis at baseline with regular follow-up were analyzed. A Kaplan-Meier analysis and a log-rank test were conducted to identify the association between exercise and incident osteoporosis in this subgroup of subjects. Event-free survival time was defined as the interval between the date of enrollment and the date of incident osteoporosis development or the last date of follow-up. All statistical analyses were performed using R version 3.6.2 and SPSS 20.0. In the study, a value of *p* < 0.05 was considered to be statistically significant.

## Results

In the cross-sectional cohort, there were 30,046 postmenopausal women enrolled in the present study with a mean age of 59 ± 5 years old. The mean menopausal age was 51 years old. There were a total of 16,806 women (56%) in the regular exercise group and 13,240 (44%) in the no exercise group (Table 1). Women with regular exercise tended to be older, with lower BMI, lower smoking rate, higher prevalence of hormone replacement therapy, lower serum fasting glucose, triglycerides, LDL cholesterol, and higher *t*-scores than those in the no exercise group (Table 1).

**Table 1:** General characteristics of postmenopausal women in the cross-sectional cohort (*N* = 30,046).

**Characteristics**	**Total**	**Regular exercise**	**No regular exercise**	***P*** **-value**
	**(*N* = 30,046)**	**(*N* = 16,806)**	**(*N* = 13,240)**	
**Demographic data**				
Age, yr	59 ± 5	60 ± 5	58 ± 5	<0.001
Age of menopause, yr	51 ± 3	51 ± 3	51 ± 3	<0.001
BMI, kg/m^2^	23.7 ± 3.5	23.5 ± 3.3	24.0 ± 3.7	<0.001
Smoke, ever, *n* (%)	1,861 (6)	811 (5)	1,050 (8)	<0.001
Alcohol status, ever, *n* (%)	715 (2)	380 (2)	335 (3)	0.137
Hormone therapy, yes, *n* (%)	1,247 (4)	792 (5)	455 (3)	<0.001
Married, yes, *n* (%)	28,428 (95)	16,030 (95)	12,398 (94)	<0.001
Education status, *n* (%)				0.767
≦Elementary	3,728 (12)	2,088 (13)	1,640 (12)	
Middle to High school	15,163 (51)	8,451 (50)	6,712 (51)	
≧Collage	11,155 (37)	6,267 (37)	4,888 (37)	
Systolic BP, mm Hg	124 ± 19	124 ± 19	123 ± 19	<0.001
Diastolic BP, mm Hg	73 ± 11	73 ± 10	73 ± 11	0.004
**Comorbidities**				
Hypertension, *n* (%)	4,764 (16)	2,715 (16)	2,049 (16)	0.112
Diabetes mellitus, *n* (%)	1,981 (7)	1,097 (7)	884 (7)	0.607
Hyperlipidemia, *n* (%)	3,245 (11)	1,885 (11)	1,360 (10)	0.009
Gout, *n* (%)	255 (1)	134 (1)	121 (1)	0.282
**Laboratory data**				
White blood counts, 10^9^/L	5.5 ± 1.5	5.5 ± 1.4	5.6 ± 1.5	<0.001
Red blood counts, 10^12^/L	4.6 ± 0.4	4.6 ± 0.4	4.6 ± 0.4	0.001
Platelet counts, 10^9^/L	235 ± 56	232 ± 55	238 ± 57	<0.001
Hemoglobin, g/dl	13.3 ± 1.0	13.3 ± 1.0	13.3 ± 1.0	0.027
Albumin, g/dl	4.5 ± 0.2	4.5 ± 0.2	4.5 ± 0.2	0.125
Fasting Glucose, mg/dl	98 ± 21	97 ± 19	98 ± 23	<0.001
Hemoglobin A1c, %	5.9 ± 0.8	5.9 ± 0.7	5.9 ± 0.9	<0.001
Total cholesterol, mg/dl	208 ± 36	208 ± 35	209 ± 37	0.109
Triglyceride, mg/dl	115 ± 76	111 ± 73	120 ± 81	<0.001
HDL cholesterol, mg/dl	59 ± 13	59 ± 14	58 ± 13	<0.001
LDL cholesterol, mg/dl	128 ± 32	127 ± 32	129 ± 33	<0.001
Creatinine, mg/dl	0.6 ± 0.2	0.6 ± 0.2	0.6 ± 0.3	0.134
Uric acid, mg/dL	5.1 ± 1.1	5.1 ± 1.1	5.1 ± 1.2	0.208
***T*** **-score**	−0.976 ± 1.430	−0.960 ± 1.431	−0.996 ± 1.428	0.031
**Osteoporosis**, ***n*** **(%)**	4,140 (13.8)	2,254 (13.4)	1,886 (14.2)	0.038

*BMI, body mass index; BP, blood pressure; HDL, high-density lipoproteins, LDL, low-density lipoproteins*.

There were 4,140 subjects (14%) having osteoporosis in the present study, 2,254 women (13%) in the regular exercise group, and 1,886 women (14%) in the no exercise group (Table 1). In the univariable binary logistic analysis, subjects with higher BMI, higher serum uric acid levels, and regular exercise had lower odds of osteoporosis. Women in the regular exercise group were associated with an ~7% decrease in the prevalence of osteoporosis s compared to those in the no exercise group [odds ratio (OR), 0.93; 95% confidence interval (95% CI), 0.87–0.97, *p* = 0.038; Table 2].

**Table 2:** Odds of osteoporosis at baseline in the cross-sectional cohort (*N* = 30,046).

**Parameters**	**Univariate analysis**		**Multivariate analysis**	
	**Odds ratio (95% CI)**	***p***	**Odds ratio (95% CI)**	***p***
Age (per 1 year)	1.095 (1.008–1.102)	<0.001	1.098 (1.090–1.107)	<0.001
Age of menopause (per 1 year)	0.991 (0.980–1.001)	0.078	-	-
Body mass index (per 1 kg/m^2^)	0.915 (0.905–0.925)	<0.001	0.908 (0.896–0.919)	<0.001
Smoke status, ever (vs. never)	0.866 (0.751–0.999)	0.049	1.061 (0.916–1.230)	0.429
Alcohol status, ever (vs. never)	1.030 (0.832–1.275)	0.785	-	-
Hormone therapy, yes (vs. no)	1.051 (0.894–1.236)	0.547	-	-
Married, yes (vs. no)	0.968 (0.838–1.117)	0.653	-	-
Education status, ≧collage (vs. others)	0.815 (0.776–0.856)	<0.001	0.837 (0.795–0.881)	<0.001
Systolic blood pressure (per 1 mmHg)	1.003 (1.001–1.005)	<0.001	1.001 (0.998–1.004)	0.440
Diastolic blood pressure (per 1 mmHg)	0.994 (0.991–0.997)	<0.001	1.003 (0.998–1.008)	0.223
Hypertension, yes (vs. no)	0.974 (0.890–1.006)	0.569	-	-
Diabetes mellitus, yes (vs. no)	0.969 (0.847–1.107)	0.639	-	-
Dyslipidemia, yes (vs. no)	0.994 (0.894–1.105)	0.909	-	-
Gout, yes (vs. no)	0.897 (0.618–1.301)	0.567	-	-
White blood counts (per 10^9^/L)	0.964 (0.942–0.986)	0.002	1.017 (0.992–1.043)	0.193
Red blood counts (per 10^12^/L)	0.662 (0.610–0.718)	<0.001	0.899 (0.825–0.979)	0.014
Platelet counts (per 10^9^/L)	0.998 (0.998–0.999)	<0.001	0.999 (0.998–1.000)	0.010
Hemoglobin (per 1 g/dl)	0.832 (0.807–0.859)	<0.001	0.886 (0.854–0.919)	<0.001
Albumin (per 1 g/dl)	0.523 (0.449–0.610)	<0.001	0.763 (0.647–0.901)	0.001
Fasting glucose (per 1 g/dl)	0.999 (0.997–1.001)	0.295	-	-
Hemoglobin A1c (per 1 %)	0.961 (0.920–1.003)	0.071	-	-
Total cholesterol (per 1 mg/dl)	0.999 (0.998–1.000)	0.100	-	-
Triglyceride (per 1 mg/dl)	0.999 (0.998–0.999)	<0.001	1.001 (1.000–1.002)	0.065
HDL cholesterol (per 1 mg/dl)	1.008 (1.006–1.010)	<0.001	1.002 (0.999–1.005)	0.151
LDL cholesterol (per 1 mg/dl)	0.998 (0.997–0.999)	<0.001	1.001 (1.000–1.002)	0.196
Creatinine, mg/dl	0.950 (0.815–1.107)	0.509	-	-
Uric acid (per 1 mg/dl)	0.839 (0.814–0.865)	<0.001	0.897 (0.867–0.927)	<0.001
Regular exercise, yes (vs. no)	0.932 (0.873–0.996)	0.038	0.760 (0.709–0.814)	<0.001

*BP, blood pressure; HDL, high-density lipoproteins; LDL, low-density lipoproteins; CI, confidence interval*.

*Multivariable model: adjustment for age, body mass index, smoke status, education status, systolic blood pressure, diastolic blood pressure, white blood counts, red blood counts, platelet counts, hemoglobin, triglyceride, low-density lipoprotein cholesterol, high-density lipoprotein cholesterol, serum albumin, and serum uric acid*.

After adjusting for confounders [using a threshold of *p* < 0.05 from the univariate analysis (Table 2)], such as age, BMI, smoking status, education status, systolic blood pressure, diastolic blood pressure, white blood counts, red blood counts, platelet counts, serum hemoglobin, albumin, triglyceride, HDL cholesterol, LDL cholesterol, and uric acid, subjects in the regular exercise group were still significantly associated with a lower prevalence of osteoporosis than those in the no exercise group (OR, 0.76; 95% CI, 0.71–0.81, *p* < 0.001; Table 2). To further examine the association between the time of each exercise and osteoporosis, a subgroup of postmenopausal women with adequate information was collected. In multivariate logistic regression analysis, women with >1 h each time had 30% lower odds of prevalent osteoporosis when compared with those in the no exercise group (Table 3).

**Table 3:** Odds of osteoporosis at baseline in the cross-sectional cohort according to the time of exercise (*N* = 29,775, excluding 271 subjects without data at hours per exercise period).

**Variables**	**No. of prevalent**	**Multivariate analysis**,	***P***
	**osteoporosis /**	**Odds ratio (95% CI)**	
	**No. of subjects (%)**		
No exercise	1,886 / 13,240 (14.2)	1.00 (Reference)	
≦1.0 h each time	1,916 / 12,257 (13.5)	0.77 (0.72–0.83)	<0.001
>1.0 h each time	290 / 2,072 (12.3)	0.68 (0.59–0.78)	<0.001

*CI, confidence interval*.

*Multivariable model: adjustment for age, body mass index, smoke status, education status, systolic blood pressure, diastolic blood pressure, white blood counts, red blood counts, platelet counts, hemoglobin, triglyceride, low-density lipoprotein cholesterol, high-density lipoprotein cholesterol, serum albumin, and serum uric acid*.

**Figure 2:**
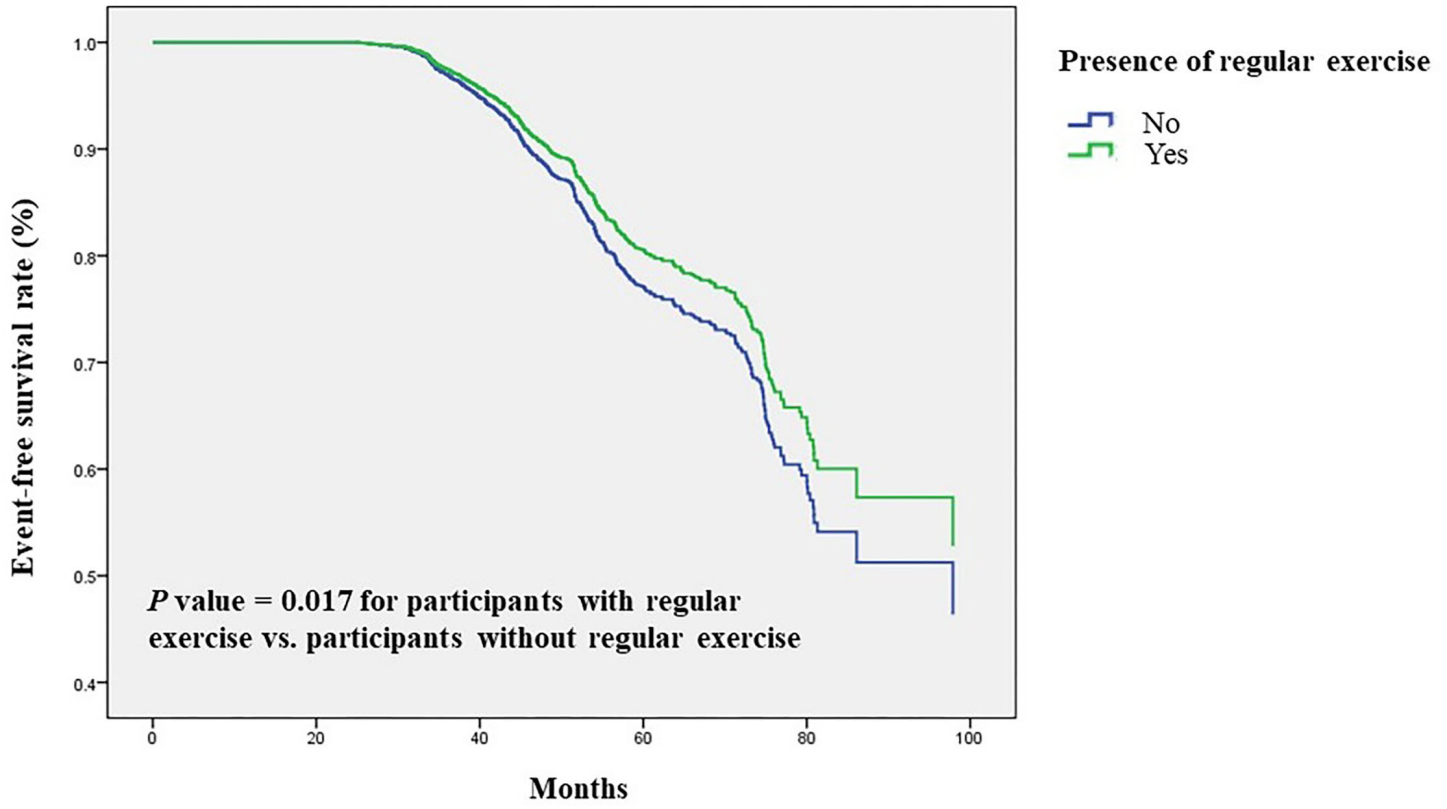
Time to osteoporosis development was longer in participants with regular exercise than in participants without regular exercise. Kaplan-Meier plot of incident osteoporosis development according to the presence of regular exercise in 6,785 participants with follow-up data.

Next, we validated our results in a longitudinal cohort of 6,785 postmenopausal women with no osteoporosis at baseline to evaluate the preventive impact of exercise on the development of osteoporosis. Of all participants, 61% had a habit of regular exercise (Table 4). During a mean follow-up period of 45 months, osteoporosis was occurred in 729 participants (10.7%). Among the regular exercise group and no exercise group, 430 subjects (10.5%) and 299 subjects (11.2%) had developed osteoporosis, respectively. In multivariate Cox regression analysis, the risk for the development of osteoporosis was significantly lower in women with regular exercise than those without exercise [hazard ratio (HR), 0.83; 95% CI, 0.71–0.97, *p* = 0.017; Table 5]. Women with longer time of exercise (>1.0 h each exercise) had a lower risk of developing osteoporosis when compared with no exercise (HR, 0.79; 95% CI, 0.66–0.95, *p* = 0.012; Table 5). The Kaplan-Meier plots of incident osteoporosis development according to the presence of regular exercise and time of exercise are shown in Figure 2. The time to osteoporosis development was longer in participants with regular exercise than in participants without regular exercise (*p* = 0.017).

**Table 4:** General characteristics of postmenopausal women in the longitudinal cohort (*N* = 6,785).

**Characteristics**	**Total**	**Regular exercise**	**No regular exercise**	***P*** **-value**
	**(*N* = 6,785)**	**(*N* = 4,112)**	**(*N* = 2,673)**	
**Demographic data**				
Age, yr	58 ± 5	59 ± 5	57 ± 5	<0.001
Age of menopause, yr	51 ± 3	51 ± 3	51 ± 3	0.004
BMI, kg/m^2^	23.9 ± 3.3	23.6 ± 3.2	24.2 ± 3.6	<0.001
Smoke, ever, *n* (%)	294 (4)	140 (3)	154 (6)	<0.001
Alcohol status, ever, *n* (%)	138 (2)	78 (2)	60 (2)	0.334
Hormone therapy, yes, *n* (%)	1,473 (4)	943 (5)	530 (4)	<0.001
Married, yes, *n* (%)	6,520 (96)	3,950 (96)	2,570 (96)	0.898
Education status, *n* (%)				0.444
≦Elementary	994 (15)	589 (14)	405 (15)	
Middle to High school	3,781 (56)	2,285 (56)	1,496 (56)	
≧Collage	2,010 (29)	1,238 (30)	772 (29)	
Systolic BP, mm Hg	121 ± 18	121 ± 18	121 ± 18	0.223
Diastolic BP, mm Hg	72 ± 10	72 ± 10	72 ± 10	0.575
**Comorbidities**				
Hypertension, *n* (%)	1,119 (17)	689 (17)	430 (16)	0.482
Diabetes mellitus, *n* (%)	458 (7)	274 (7)	184 (7)	0.729
Hyperlipidemia, *n* (%)	691 (10)	417 (10)	274 (10)	0.902
Gout, *n* (%)	64 (1)	32 (1)	32 (1)	0.094
**Laboratory data**				
White blood counts, 10^9^/L	5.7 ± 1.4	5.6 ± 1.4	5.8 ± 1.5	<0.001
Red blood counts, 10^12^/L	4.6 ± 0.4	4.6 ± 0.4	4.6 ± 0.4	0.133
Platelet counts, 10^9^/L	236 ± 53	234 ± 53	240 ± 54	<0.001
Hemoglobin, g/dl	13.3 ± 1.0	13.3 ± 1.0	13.4 ± 1.0	0.032
Albumin, g/dl	4.5 ± 0.2	4.5 ± 0.2	4.5 ± 0.2	0.932
Fasting Glucose, mg/dl	97 ± 20	97 ± 18	98 ± 23	0.006
Hemoglobin A1c, %	5.9 ± 0.8	5.9 ± 0.7	5.9 ± 0.9	0.003
Total cholesterol, mg/dl	208 ± 35	208 ± 35	208 ± 36	0.642
Triglyceride, mg/dl	115 ± 73	112 ± 68	120 ± 79	<0.001
HDL cholesterol, mg/dl	58 ± 13	59 ± 13	57 ± 13	<0.001
LDL cholesterol, mg/dl	128 ± 32	128 ± 32	130 ± 33	0.008
Creatinine, mg/dl	0.6 ± 0.2	0.6 ± 0.2	0.6 ± 0.2	0.239
Uric acid, mg/dL	5.2 ± 1.2	5.2 ± 1.1	5.2 ± 1.2	0.007
**Follow-up, months**	45.2 ± 12.8	45.0 ± 12.8	45.5 ± 12.7	0.076

*BMI, body mass index; BP, blood pressure; HDL, high-density lipoproteins; LDL, low-density lipoproteins*.

**Table 5:** The relative risk for incident osteoporosis in the longitudinal cohort according to the presence of regular exercise (*N* = 6,785) and time of exercise (*N* = 6,769, excluding 16 subjects without data at hours per exercise period).

**Variables**	**No. of incident**	**Adjusted Hazard**	***P*** **-value**
	**osteoporosis /**	**Ratio (95% CI)**	
	**No. of subjects (%)**		
**Regular exercise (yes vs. no)**			
No	299 / 2,673 (11.2)	1.000 (Reference)	
Yes	430 / 4,112 (10.5)	0.83 (0.71–0.97)	0.017
**Time of exercise**			
No exercise	299 / 2,673 (11.2)	1.000 (Reference)	
≦1.0 h each time	210 / 1,985 (10.6)	0.87 (0.73–1.04)	0.130
>1.0 h each time	215 / 2,111 (10.2)	0.79 (0.66–0.95)	0.012

*CI, confidence interval*.

*Multivariable model: adjustment for age, body mass index, smoke status, education status, systolic blood pressure, diastolic blood pressure, white blood counts, red blood counts, platelet counts, hemoglobin, triglyceride, low-density lipoprotein cholesterol, high-density lipoprotein cholesterol, serum albumin, and serum uric acid*.

## Discussion

In this cross-sectional and longitudinal study of a large-scale, community-based, representative female population in Taiwan, regular exercise was associated with a reduced risk of osteoporosis in postmenopausal women after adjustment for confounders. To the best of our knowledge, this study is the largest community-based investigation to verify the preventive effect of regular physical exercise on the development of osteoporosis in postmenopausal women. Moreover, a dose-response relationship between the time of exercise and the risk of osteoporosis was identified and suggested that the longer the exercise, the lower the risk for developing osteoporosis.

According to the World Health Organization (WHO), musculoskeletal conditions, such as low back pain and osteoporosis, are the main causes of disability worldwide and the greatest contributors to the global need for rehabilitation (29). Women are at greater risk for developing osteoporosis than men because the peak bone mass at skeletal maturity (from 30 to 35 years of age) in women is on average 30% lower than that in men and, moreover, rapid decline in bone mass occurs due to estrogen withdrawal during and after menopause (30). Menopause overall leads to a yearly average of bone loss of 1–1.5% throughout postmenopausal years, such as a more rapid bone loss of >2–3% during the initial 6–10 years and 0.5–1% thereafter (31–33), contributing to the greater incidence of osteoporosis and consequent fracture in postmenopausal women. There is an urgent need to develop a way to reduce osteoporosis, fractures, and loss of BMD in this group of people. Our present study suggests an association between exercise and reduction in osteoporosis in postmenopausal women and may inform future prevention strategies.

Previously, several studies have been conducted concerning exercise and osteoporosis among the elderly and postmenopausal women. A large community-based study in a cohort of California adults with a mean age of 73 years (1,014 women and 689 men) suggests a protective effect of exercise on hip BMD (34). Similarly, another cross-sectional, community study in Southern Europe examined the relationship between a questionnaire-based exercise report and spine BMD in postmenopausal women (*N* = 1,373) and the results showed that participants in the low exercise group (defined as exercise < 2 h per week) had higher odds of osteoporosis (OR, 1.67; 95% CI, 1.06–2.64) when compared with those in the high exercise group (defined as ≧ 5 h per week) (35). In line with these studies, our large-scale, cross-sectional, and longitudinal epidemiologic survey has observed that regular exercise is associated with a reduced risk of osteoporosis in postmenopausal women.

Based on these community-based studies, numerous experimental studies were also designed to evaluate exercise and osteoporosis prevention in public health programs. A Brazil study randomly assigned 25 postmenopausal women into trained and untrained groups and found that the untrained women had significantly greater reductions in BMD than those in the trained group (19). Similar results were also noted in another randomized controlled trial by Iwamoto et al., who reported that exercise could lead to a significant increase in BMD in postmenopausal women when compared with women without exercise (20). Moreover, they also found that the BMD in women with detraining would revert to a level comparable to those without exercise, which highlights the importance of continuous exercise (20). A systemic review and meta-analysis, i.e., 43 randomized controlled trials and 4,320 participants, also showed that exercise can improve BMD and reduce the chances of fractures in postmenopausal women (36).

However, two other randomized controlled trials in Sweden and America found that postmenopausal women who exercised did not experience significant increases in BMD (37, 38). In Sweden, Bergström et al. enrolled 120 postmenopausal women with forearm fractures and randomized them into training and control groups. They found that there were no significant differences in BMD between the two groups during a 1-year follow-up period (37). An American study compared the effects of a high-load and a high-repetition physical training program on muscular strength and BMD in menopausal women during a 6-month period. The results showed that physical training improved muscular strength, but no improvements in BMD were observed (38). Both studies had small participants and short follow-up durations, furthermore, different training protocols, types of subjects, and diversity in methodology could potentially contribute to the variability of exercise and its inconsistent association with BMD.

A strength of the present study is that we demonstrated the dose-response effects of exercise on the reduction of incident osteoporosis. In our study, postmenopausal women with longer hours of exercise were significantly associated with a decrease in developing osteoporosis when compared with shorter hours of exercise. A recent clinical trial enrolled 400 postmenopausal women and randomized them to either moderate (150 min per week) or high volume (300 min per week) exercise groups, and they found that higher volumes of exercise lead to a smaller decline in BMD during a 24-month follow-up (14). A systemic review and meta-analysis also suggested a positive effect of high volume exercise on bone health (39). Although these studies have different exercise protocols, it can still be found that the longer the exercise, the more effective the prevention of osteoporosis.

In the present study, we demonstrated a large-scale, community-based, and multiple-covariate survey among more than 30,000 postmenopausal women to clarify the positive association between regular exercise and the prevention of osteoporosis. Additionally, exercise time was also investigated to certify the dose-response effect of exercise. Despite these strengths, there were several limitations. The first limitation was the method of measurement used in BMD. Although dual-energy X-ray absorptiometry (DXA) is a standard method for the diagnosis of BMD, the equipment is very expensive and not easily available or accessible for BMD measurement for a large number of examinees (11, 40). Quantitative ultrasound measurement, typically at the heel calcaneus, is an alternative method of estimating bone mass based on the strength of its moderate to high correlations with DXA-derived BMD (41, 42). Furthermore, the ultrasound measurement used in the current study has the advantage of being relatively inexpensive, quick, and safe and thus, is beneficial in assessing very large sample sizes in practice (43, 44). Secondly, the database at TWB was short on information on the specific types, intensity, and time (duration) of habitual exercise implemented by our examinees. A third limitation was the use of self-administered questionnaires to inquire about habitual exercise rather than strict administration of specific exercise interventions to investigate the effects of specific exercise interventions on bone health. Fourthly, there were no data on the medical record of fractures during follow-up, which limited the understanding of the effects of exercise on the decrease in risk of osteoporotic fractures. Fifthly, all the examinees came from Taiwan, which might limit our ability to generalize a decisive conclusion from our findings. Sixthly, the volunteers in TWB were between 30 and 70 years old, and we focused only on postmenopausal women, meaning the majority of the study cohort was between 55 and 65 years of age. This may limit our interpretation of the proportion of women who were physically active in different age groups. Lastly, other variables, such as dietary habits and nutrient supplementation (such as calcium, vitamin D, and protein intake), remain to be investigated to clarify their influence on the prevention of osteoporosis in postmenopausal women.

## Conclusion

Our community-based, large-scale longitudinal study demonstrated that regular exercise was strongly associated with a decrease in incident osteoporosis in postmenopausal women, and a longer time of exercise (>1 h) displayed significantly greater preventive efficacy for postmenopausal osteoporosis. It is suggested that healthcare professionals, such as physical therapists, should encourage postmenopausal women to exercise regularly to improve their bone health, prevent osteoporosis, avert subsequent, and prevent extremely harmful complications, such as fractures.

## Data Availability Statement

The dataset in from third party institute and could be apply only under reasonable access. Requests to access these datasets should be directed to mi0909099@ibms.sinica.edu.tw.

## Ethics Statement

The studies involving human participants were reviewed and approved by the Institutional Review Board of Kaohsiung Medical University Hospital (KMUHIRB-E(I)-20210058). The patients/participants provided their written informed consent to participate in this study.

## Author Contributions

J-HG and C-FC: conception and design. J-HG, J-IL, and S-CC: acquisition of data. C-FC, J-HG, and S-PH: drafting of the manuscript. J-HG and J-IL: statistical analysis. J-IL, S-CC, S-PH, and J-HG: administrative, technical, or material support. All authors: analysis and interpretation of data and critical revision of them anuscript for important intellectual content. All authors contributed to the article and approved the submitted version.

## Funding

This work was supported partially by the Research Center for Environmental Medicine, Kaohsiung Medical University, Kaohsiung, Taiwan, from the Featured Areas Research Center Program within the framework of the Higher Education Sprout Project by the Ministry of Education (MOE) in Taiwan, and by Kaohsiung Medical University Research Center Grant (KMUTC109A01-1 and KMUTC111IFSP01) and by Tzu Chi University.

## Conflict of Interest

The authors declare that the research was conducted in the absence of any commercial or financial relationships that could be construed as a potential conflictof interest.

## Publisher's Note

All claims expressed in this article are solely those of the authors and do not necessarily represent those of their affiliated organizations, or those of the publisher, the editors and the reviewers. Any product that may be evaluated in this article, or claim that may be made by its manufacturer, is not guaranteed or endorsed by the publisher.
